# Different approaches to the treatment of skeletal Class II malocclusion during growth: Bionator versus extraoral appliance

**DOI:** 10.1590/2177-6709.25.2.069-085.bbo

**Published:** 2020

**Authors:** Renato Barcellos Rédua

**Affiliations:** 1 Private practice, Vittória/ES, Brazil

**Keywords:** Activating appliances, Extraoral traction appliances, Angle Class II malocclusion

## Abstract

**Introduction:**

Class II malocclusion, which has a significant incidence in the population, may compromise facial esthetics and the smile, as well as the masticatory and respiratory functions. Often associated with skeletal abnormalities, it severely affects and compromises quality of life. An accurate diagnosis is fundamental to prepare a treatment plan to correct dental and skeletal anomalies.

**Objectives:**

This study discusses treatment alternatives to the correction of Class II division 1 and 2 malocclusion in growing patients, using a Bionator and an extraoral appliance.

## RESUMO

**Introdução:**

A má oclusão de Classe II apresenta uma incidência significativa na população, podendo comprometer a estética facial, o sorriso e as funções mastigatória e respiratória. Frequentemente está associada à alteração esquelética, o que aumenta a repercussão desse comprometimento, interferindo ainda mais na qualidade de vida das pessoas afetadas. O correto diagnóstico dessa condição é fundamental para a elaboração de um plano de tratamento que permita a correção dentária e esquelética.

**Objetivos:**

O objetivo do presente artigo é discutir as alternativas terapêuticas para correção da Classe II divisão 1 e 2 em pacientes em fase de crescimento, utilizando-se o Bionator de Balters ou o aparelho extrabucal.

## INTRODUCTION

Class II malocclusion, the distal relationship between mandibular and maxillary molars^1^, is very frequent in the population. It affects 22.6% of the American children aged 8 to 11 years^2^, 28% of the Dutch population^3^, 23% of the Colombian children 5 to 17 years old^4^, 19% of the Lebanese^5^, and 38% of the Brazilian children 7 to 12 years old^6^, with no sex predilection.

Class II malocclusion may be associated with skeletal abnormalities in about 75% of the patients^7^, who usually present with characteristic mandibular retrognathism resulting from a shortened mandible^8^ and maxillary protrusion. 

Dental and skeletal Class II malocclusion carries a greater risk of dental trauma^10^, a more negative perception of facial^11^ and dental^12^ esthetics, a negative impact on quality of life and self-esteem^13^, a greater predisposition to periodontal diseases^14^ and tooth wear^15^ ^,^ ^16^, and a reduction of oropharyngeal space and greater incidence of sleep disorders^17^. 

The advantage of treating Class II malocclusion during growth, that is, in the mixed or early permanent dentition stage, is the possibility of changing the patient's growth pattern^18^ ^-^ ^20^ and reducing the risk of trauma to maxillary incisors. In addition, it increases airway space in the oropharyngeal region and results in an ideal and stable occlusion^18^. 

Several treatment options have been described in the literature for the correction of dental and skeletal Class II malocclusion in growing patients, such as: 1) a two-stage treatment using a functional appliance in the first stage and a fixed appliance in the second^18^; 2) a one-stage treatment using an extraoral appliance combined with a fixed appliance^18^; and 3) the use of a mandibular fixed protraction appliance, such as Herbst and Forsus, before or at the same time as a fixed appliance^20^. This study discusses the results of two types of treatment of skeletal Class II malocclusion, using a Balters Bionator or an extraoral appliance. 

The Bionator, developed by Wilhelm Balters in the 1950s, is a removable functional orthopedic activator that acts on both the orofacial muscle positioning and the primary anterior displacement of the mandible. It is used to correct Class II malocclusion by means of stimulation or acceleration of mandibular growth, combined with maxillary growth restriction and anterior displacement^22^ ^-^ ^24^. In addition, maxillary incisors are retroclined, the mandibular incisors, proclined, overjet is reduced and the Angle Class II molar relationship is corrected^17^ ^,^ ^20^ ^,^ ^23^ ^,^ ^24^. 

Patient cooperation is fundamental, because the appliance is removable and cannot be used together with a fixed appliance. In the beginning, the patient may undergo changes in speech and social routines, but will be adapted to them in a few weeks. 

The maxillary extraoral traction appliance, developed by Norman Willian Kingsley in 1866, was the precursor of innumerable mechanical devices later developed by Angle^25^, Tweed-Merrifield^26^, Thurow^27^ and Graber^28^, among others. Its mechanism of action consists of the correction of dental and skeletal Class II malocclusion by redirecting maxillary growth and moving maxillary teeth distally. The posterior region of the head is the site of anchorage for the application of forces, which may vary in direction - cervical, parietal or combined - to control vertical maxillary growth, and in amount of force, to produce orthopedic or orthodontic results^18^ ^,^ ^24^ ^-^ ^26^. Orthopedic forces may be applied directly on the maxillary permanent molar or on the removable appliance adapted to the maxillary arch. As it is also a removable appliance, treatment success again depends on patient cooperation. It may affect the patient’s routine, depending on the type of protocol followed. A total of 12 to 14 hours a day, including sleeping hours, is enough to obtain excellent results in patients during pubertal growth spurt. This use frequency has a very little effect on any individual's routine. This appliance does not affect speech, and its use may be combined with that of a fixed appliance to increase treatment efficacy. Extraoral forces have been successfully indicated to correct discrepancies between the dental arches and teeth, both in Class II and Class III malocclusion. The different types of extraoral appliances used on the dental arches and structures of the craniofacial complex have been extensively discussed in the literature. 

Two clinical cases of Class II malocclusion and different treatment options are reported below. 

## CLINICAL CASE REPORTS

### Clinical Case 1 - Treatment of Class II division 1 malocclusion using Balters Bionator

A Caucasian 10-year and 7-month-old girl in the second stage of mixed dentition was dissatisfied with not being able to close her mouth and with her “bucktoothed” appearance. Medical and dental history did not reveal any important information about tooth integrity, respiratory problems or the presence of snoring or sleep apnea. 

**Figure 1:**
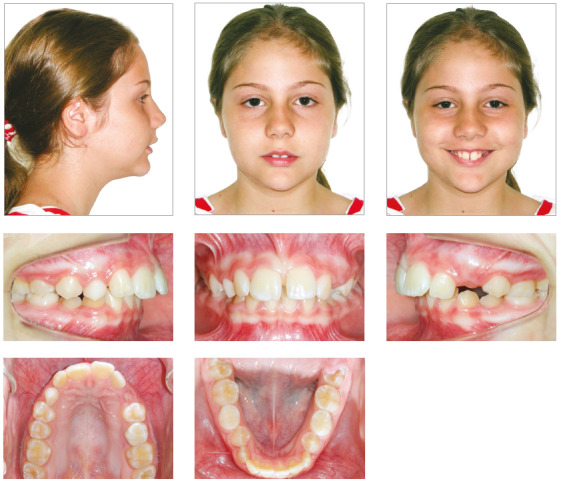
Case 1: Baseline facial and intraoral photographs.

Facial analysis revealed frontal face symmetry, maxillary dental midline coincident with facial midline, and no lip seal. Functional analysis revealed adequate exposure of maxillary incisors while speaking and smiling. The lateral view showed a convex profile, due to a mild mandibular retrusion, and a normal vertical pattern. She had an obtuse nasolabial angle and an everted lower lip. There were no joint noises or symptoms of temporomandibular joint dysfunction, and no deviations during mandibular movements (Fig. 1). The analysis of occlusion revealed an Angle Class II division 1 malocclusion, 8-mm overjet, deep overbite, slight deviation of dental midlines, as the mandibular midline was slightly displaced to the right, maxillary midline diastema and no significant space discrepancies, with enough room for the successors. The patient’s oral hygiene was good, and she had no restorations or caries (Fig. 1). 

**Figure 2:**
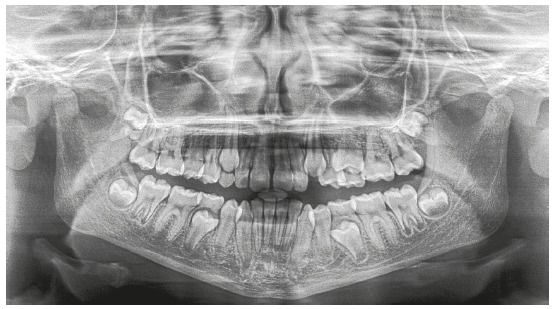
Case 1: Baseline panoramic radiograph.

The panoramic radiograph confirmed the presence of all permanent teeth, and the lateral radiograph revealed that airway space had no obstructions. The stage of cervical vertebrae maturation was compatible with the beginning of the pubertal growth spurt (Fig. 2).

**Figure 3:**
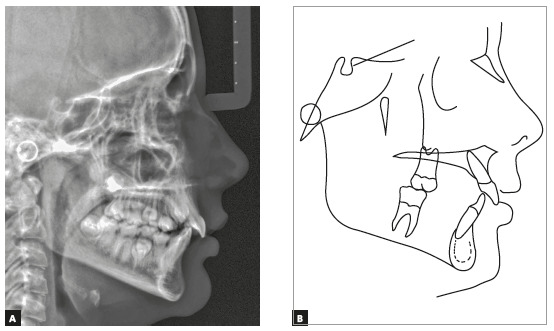
Case 1: Baseline cephalometric lateral radiograph (A) and cephalometric tracing (B).

Cephalometric analysis confirmed a Class II skeletal pattern (ANB = 6°, NAPog = 7°), mild mandibular deficiency and a horizontal growth pattern (SN.GoGn = 26°). Maxillary and mandibular incisors were in a normal position in relation to the basal bone (Fig. 3 and Table 1).

**Table 1:** Case 1: Baseline (A), intermediate (B) and final (C) cephalometric values.

	Measurements		Normal	A	B	C	A/C diff.
Skeletal pattern	SNA	(Steiner)	82°	83°	83°	82°	1
	SNB	(Steiner)	80°	77°	79°	81°	4
	ANB	(Steiner)	2°	6°	4°	1°	5
	Wits	(Jacobson)	♀ 0 ± 2mm	3mm	2mm	0mm	3
			♂ 1 ± 2mm				
	Angle of convexity	(Downs)	0°	7°	3°	1°	6
	Y-axis	(Downs)	59°	56°	59°	60°	4
	Facial angle	(Downs)	87°	89°	89°	86°	3
	SN.GoGn	(Steiner)	32°	26°	27°	30°	4
	FMA	(Tweed)	25°	19°	21°	22°	3
Dental pattern	IMPA	(Tweed)	90°	101°	101°	103°	2
	1.NA (degrees)	(Steiner)	22°	24°	20°	21°	3
	1-NA (mm)	(Steiner)	4 mm	5mm	3mm	3mm	2
	$\bar{1}$.NB (degrees)	(Steiner)	25°	27°	28°	31°	4
	$\bar{1}$-NB (mm)	(Steiner)	4 mm	4mm	4mm	5mm	1
	$\frac{1}{1}$ - Interincisal angle	(Downs)	130°	124°	128°	129°	5
	$\frac{1}{1}$ - Apo	(Steiner)	1 mm	2 mm	3 mm	2 mm	0
Profile	Upper lip - S-line	(Steiner)	0	0mm	-2mm	-3mm	3
	Lower lip - S-line	(Steiner)	0	0mm	-3mm	-2mm	2

### Treatment plan and mechanics

**Figure 4:**
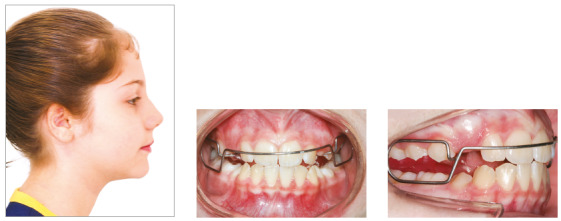
Case 1: Intermediate facial and intraoral photographs: Balters Bionator.

**Figure 5:**
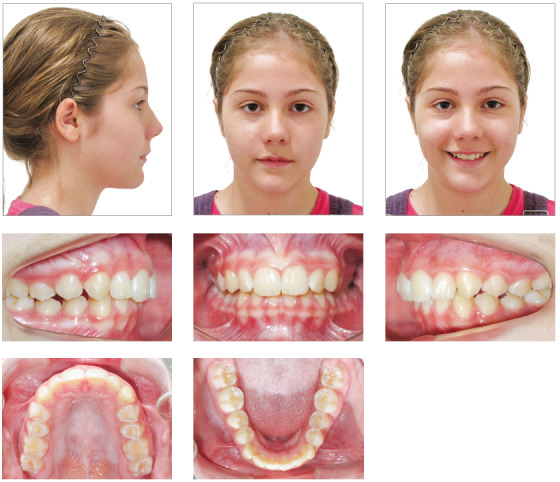
Case 1: Intermediate facial and intraoral photographs after stage 1.

**Figure 6:**
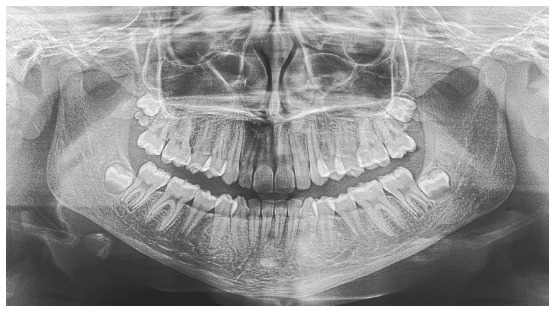
Case 1: Intermediate panoramic radiograph after stage 1.

**Figure 7:**
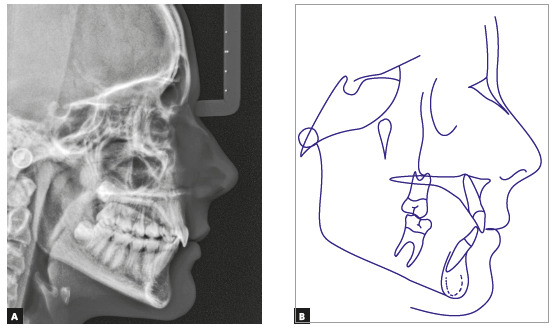
Case 1: Intermediate cephalometric lateral radiograph (A) and cephalometric tracing (B) after stage 1.

The objective of the treatment was to correct the skeletal Class II and Angle Class II division 1 malocclusion. In the first stage, a removable protraction appliance, the Balters Bionator^22^ ^,^ ^23^, was indicated to stimulate mandibular growth. It should be used all the time, except in the first month, when it should be removed for school activities, for speech adaptation. After that time, the appliance should be used for as long as possible (Fig. 4). This treatment stage lasted 11 months, and, due to good patient cooperation, facial growth pattern was changed, which contributed to reducing bone profile convexity, Class II malocclusion and overjet (Figs 5, 6 and 7). 

Seven months later, the second treatment stage began with a fixed 0.022 x 0.028-in Roth prescription Straight-wire appliance. Dental alignment and leveling of both arches were achieved using 0.014” nickel-titanium archwires and 0.016 to 0.020-in stainless steel wires. After that, intermaxillary Class II elastics and 0.019 x 0.025-in stainless steel archwires were used to achieve adequate intercuspation and to finish the correction of Class II malocclusion. The patient used a lingual 0.028-in stainless steel wire retainer and a maxillary wraparound retainer full time in the first six months and only at night for six more months. Treatment lasted 11 months in the first stage, followed by a 7-month interval, and 18 months in the second stage.

### Results

**Figure 8:**
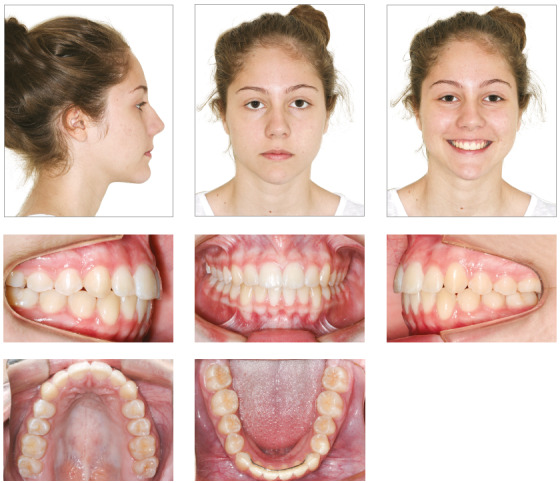
Case 1: Final facial and intraoral photographs.

**Figure 9:**
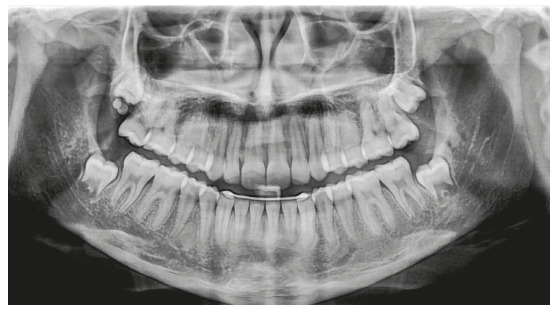
Case 1: Final panoramic radiograph.

**Figure 10:**
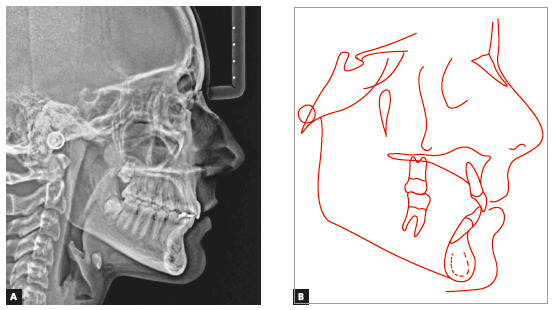
Case 1: Final cephalometric lateral radiograph (A) and cephalometric tracing (B).

**Figure 11:**
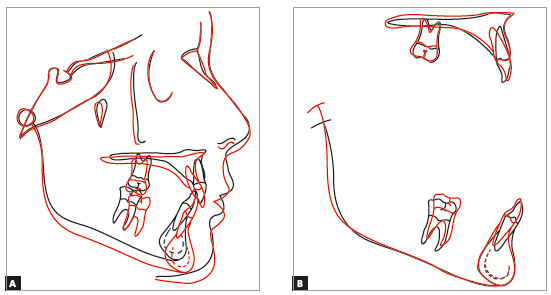
Case 1: Total (A) and partial (B) superimpositions of baseline (black) and final (red) cephalometric tracings.

**Figure 12:**
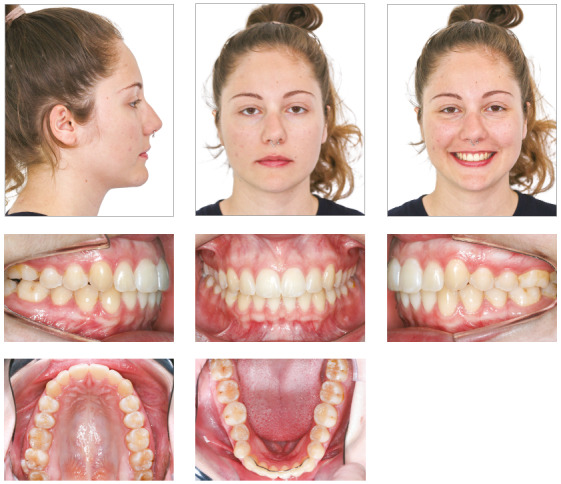
Case 1: Facial and intraoral photographs: four years after orthodontic treatment completion.

Treatment resulted in an Angle Class I molar relationship, intercuspation, adequate overjet, overbite, and bilateral canine and anterior guidance free of interferences. Dental midlines were coincident with facial midline and between each other. A straight profile and good lip seal resulted from the stimulation of mandibular growth during pubertal growth spurt (Fig 8). ANB was reduced from 6° to 3°, and the angle of convexity, from 7° to 1° (Table 1). Good periodontal health and dental integrity were preserved, and there were no root resorptions, as seen on the final panoramic radiograph (Figs 9 and 10, and Table 1)^29^. The superimposition of baseline and final cephalometric tracings revealed substantial mandibular growth in a favorable direction. There was no maxillary molar distalization, and mandibular incisors were minimally proclined (Fig 11). Four years after treatment completion, occlusive stability, periodontal health and absence of functional changes were confirmed (Fig 12).

### Clinical Case 2 - Treatment of Class II division 2 malocclusion using extraoral appliance

A white 10-year and 11-month-old girl in the second stage of mixed dentition was dissatisfied with the position of her teeth, and classified them as “crooked”. Her medical and dental history did not reveal anything relevant. 

**Figure 13:**
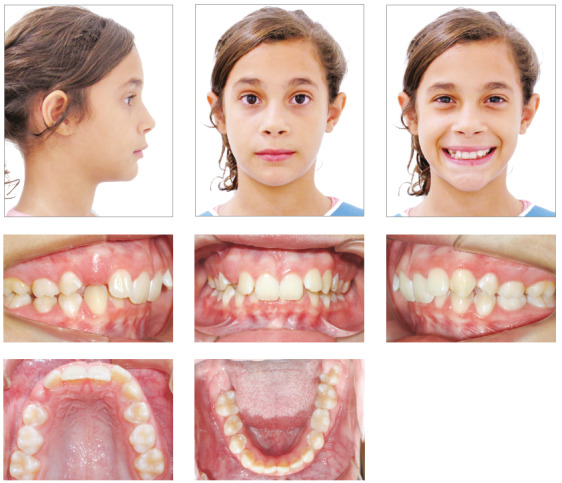
Case 2: Baseline facial and intraoral photographs.

Facial analysis revealed frontal face symmetry, good lip seal, adequate exposure of maxillary incisors when speaking and smiling, and the maxillary dental midline was coincident with the facial midline. The lateral view revealed a convex profile, discrete mandibular retrognathism, straight nasolabial angle and a balanced vertical pattern (Fig 13). There were no joint noises or symptoms of temporomandibular joint dysfunction, and no deviations during mandibular movements. Occlusal examination revealed an Angle Class II division 2 malocclusion, with retroclined maxillary central incisors and a deep overbite. Dental midlines were coincident. There was no important lack of space for the eruption of permanent teeth. The patient’s oral hygiene was good, and she had no restorations or caries (Fig 13). 

**Figure 14:**
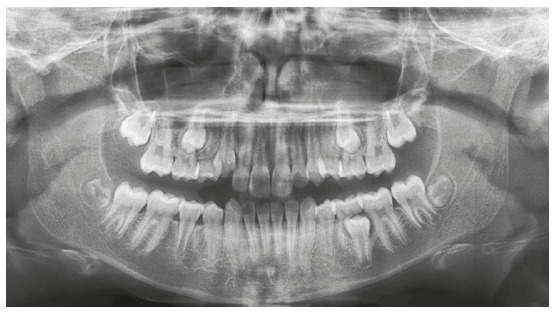
Case 2: Baseline panoramic radiograph.

**Figure 15:**
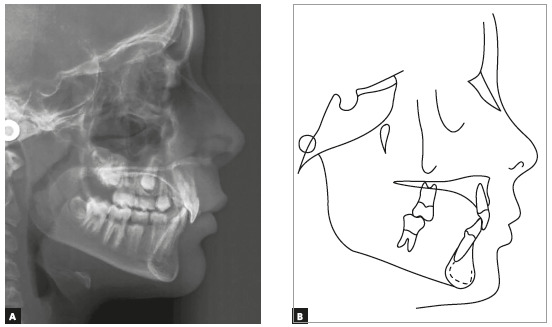
Case 2: Baseline cephalometric lateral radiograph (A) and cephalometric tracing (B).

The panoramic radiograph confirmed the presence of all permanent teeth, and the lateral radiograph showed that the upper airway space was slightly reduced, but without any obstructions. The stage of cervical vertebrae maturation was compatible with the beginning of pubertal growth spurt (Figs 14 and 15).

Cephalometric analysis confirmed skeletal Class II pattern, with mandibular retrusion (ANB = 6°, NA.Pog = 12°) and normal vertical dimensions (SN.GoGn = 32°). Maxillary incisors were retroclined (1.NA = 2° and 1-NA = 0 mm), and mandibular incisors, proclined (IMPA = 100°) (Fig 15 and Table 2). 

**Table 2:** Case 2: Baseline (A) and final (B) cephalometric values.

	Measurements		Normal	A	B	A/B diff.
Skeletal pattern	SNA	(Steiner)	82°	81°	81°	0
	SNB	(Steiner)	80°	75°	77°	2
	ANB	(Steiner)	2°	6°	4°	2
	Wits	(Jacobson)	♀ 0 ± 2mm	5mm	3mm	2
			♂ 1 ± 2mm			
	Angle of convexity	(Downs)	0°	12°	5°	7
	Y-axis	(Downs)	59°	59°	59°	0
	Facial angle	(Downs)	87°	85°	87°	2
	SN.GoGn	(Steiner)	32°	32°	29°	3
	FMA	(Tweed)	25°	21°	22°	1
Dental pattern	IMPA	(Tweed)	90°	100°	102°	2
	1.NA (degrees)	(Steiner)	22°	2°	17°	15
	1-NA (mm)	(Steiner)	4 mm	0mm	4mm	4
	1.NB (degrees)	(Steiner)	25°	26°	27°	1
	1-NB (mm)	(Steiner)	4 mm	4mm	5mm	1
	- Interincisal angle	(Downs)	130°	146°	131°	15
	- Apo	(Steiner)	1 mm	1 mm	1 mm	0
Profile	Upper lip - S-line	(Steiner)	0	3mm	2mm	1
	Lower lip - S-line	(Steiner)	0	1mm	0mm	1

### Treatment plan and mechanics

Treatment plan consisted of a one-stage treatment for Class II malocclusion using an extraoral traction appliance combined with a fixed appliance. The extraoral appliance, which applied a force of about 400 g per side, should be used at home for at least 12 h a day or longer, if possible. 

A fixed 0.022 x 0.028-in Roth prescription straight wire appliance was placed during the second stage of mixed dentition, but did not include teeth #55, #65 and #75, which were about to exfoliate. Leveling and alignment, which lasted 11 months, were conducted at the same time as molar distalization using the extraoral appliance. After leveling and alignment, 0.019 x 0.025-in stainless steel archwires and Class II intermaxillary orthodontic mechanics were used, still in combination with the extraoral appliance. The same retention protocol described in Case 1 was used. Total active treatment time was 24 months, after which the final tests and imaging studies were obtained for the evaluation of results.

## Results

**Figure 16:**
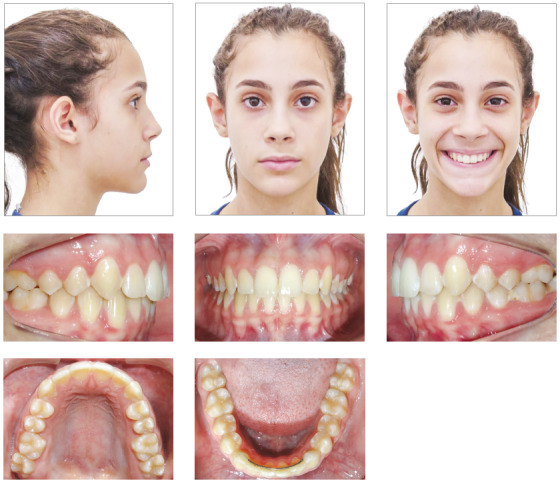
Case 2: Final facial and intraoral photographs.

**Figure 17:**
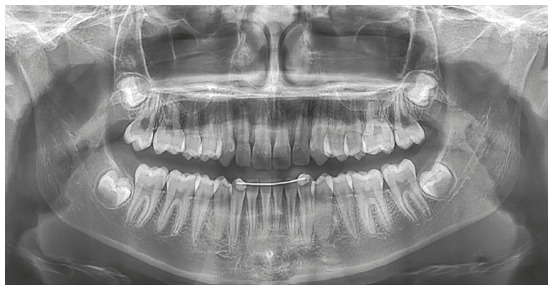
Case 2: Final panoramic radiograph.

Treatment results were satisfactory and led to an Angle Class I molar relationship, intercuspation, adequate overjet and overbite and lateral canine and anterior functional guidance free of interferences. A straight profile resulted from the change of facial growth pattern, because the treatment was conducted at the time of the patient’s pubertal growth spurt (Fig 16). ANB was reduced from 6° to 4°, and the angle of convexity, from 12° to 1° (Table 2). The final panoramic radiograph confirmed the integrity of dental structures (Fig 17). 

The superimposition of baseline and final cephalometric tracings revealed good mandibular growth in a favorable direction. Facial growth pattern changed, mainly because anterior displacement of the maxilla was contained (SNA remained 81°), while the mandible grew as expected (SNB from 75° to 77°). There was no proclination of mandibular incisors, and the retroclination of maxillary incisors was corrected (Fig. 19). Three years after treatment completion, occlusion stability, periodontal health and absence of functional changes were confirmed (Fig. 20).

**Figure 18:**
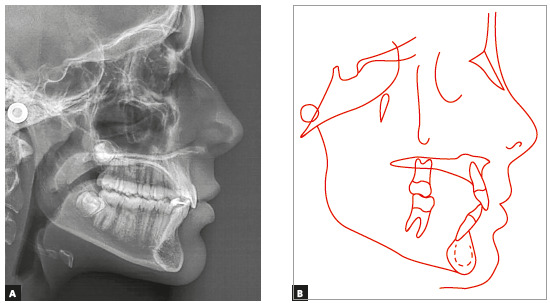
Case 2: Final cephalometric lateral radiograph (A) and cephalometric tracing (B).

**Figure 19:**
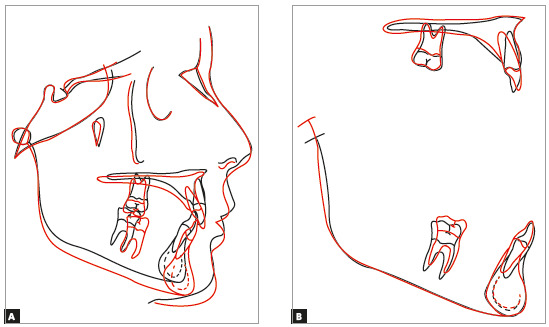
Case 2: Total (A) and partial (B) superimpositions of baseline (black) and final (red) cephalometric tracings.

**Figure 20:**
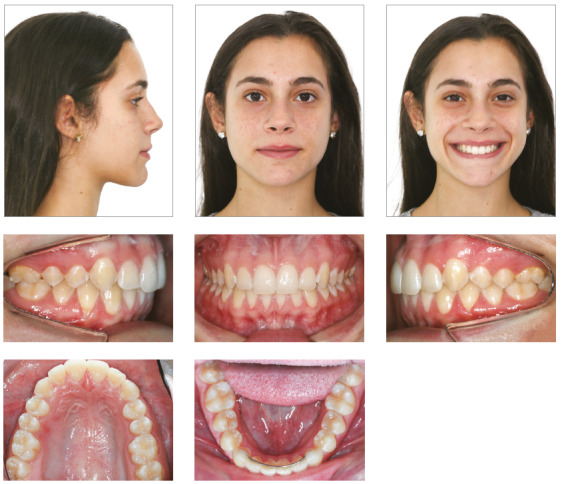
Case 2: Facial and intraoral photographs: three years after orthodontic treatment completion.

## DISCUSSION

The methods for correction of Class II malocclusion during growth described in the literature include fixed orthodontic appliances and removable aligners combined with intermaxillary elastics, extraoral appliances^18^ ^,^ ^19^ ^,^ ^26^, temporary skeletal anchorage, functional appliances and several types of fixed and removable protraction appliances^18^ ^,^ ^22^ ^,^ ^31^ ^,^ ^32^. Align Technology has recently developed a system known as “wings" for its aligners to correct Class II malocclusion by means of mandibular protraction. However, there is still no scientific evidence about this system. Of all the alternatives available, it is important to consider which ones allow the correction of skeletal Class II malocclusion. 

The Brazilian Association of Orthodontics (ABOR) and the American Association of Orthodontics (AAO) recommend that children should have their first visit to the orthodontist at about the age of seven years, that is, during the first stage of mixed dentition. Studies in the literature indicate that, in this stage, there are better orthopedic responses to correct posterior skeletal crossbite^32^, anterior open bite^33^ and skeletal Class III malocclusion^34^, as well as to monitor spaces and diagnose impactions or ectopic teeth. Several randomized controlled trials investigating the treatment of Class II malocclusion during growth had similar results for both the two-stage interventions - first at about the age of seven years and then during the time of young permanent dentition - and the one-stage treatment during pubertal growth spurt^18^ ^,^ ^11^ ^,^ ^35^. 

Class II malocclusion in Case 1 was treated in two stages, using a Balters Bionator in the first stage and a fixed appliance combined with Class II elastics in the second stage. The patient’s parents were also told that the treatment could be conducted in a single stage using an extraoral appliance combined with a fixed appliance, which would reduce the time and costs of the treatment. Her patents were also aware that both approaches would have similar results.^18^ ^,^ ^36^ Their choice of a two-stage treatment was based on the fact that it did not require the use of an extraoral appliance. Nowadays, school, sports and social activities of children and, above all, adolescents should be taken into consideration when making treatment plans. The use of an extraoral appliance outside the home may result in embarrassment. Bionator, an intraoral appliance, may lead to fewer adverse reactions in social life, but the changes in speaking that result from its use may also be a source of embarrassment. 

In Case 2, Class II malocclusion was corrected using an extraoral appliance for at least 12 hours a day, as this is the shortest time to achieve satisfactory results. The clinical results of the two cases described above were directly associated with patient adherence to the treatment, regardless of whether it had one or two stages. 

In both cases, there was patient cooperation. The consequent changes in their facial pattern were assigned to the orthopedic result of orthodontic mechanics and favorable mandibular growth. This was true even of Case 2, in which no appliance was used to stimulate growth. Treatments were conducted during the time of greatest mandibular response to orthopedic interventions, that is, during the pubertal growth spurt. Therefore, the duration of the treatment with an extraoral appliance was shortened, as the skeletal correction was performed at the same time as dental leveling and alignment.

The best time and approach to treat Class II malocclusion in one or two stages has been extensively investigated in Orthodontics. Systematic reviews in the literature provide the best scientific evidence, and one of the sources for them is the Cochrane Library^37^. One of these reviews published at Cochrane Library concluded that early treatment of Class II malocclusion and maxillary incisor protrusion in adolescence was not more efficient than treatment in a single stage in the beginning of puberty^38^. Randomized clinical trials conducted in the United States and England^18^ ^,^ ^39^ also concluded that, after the second stage of the treatment of Class II malocclusion, there were no differences between the group submitted to early intervention and the group treated in a single stage later. Therefore, the choice of a two- or one-stage treatment seems to be a matter of professional preference, rather than a biological decision. 

In the two cases described here, a straight and harmonious profile was obtained at the end of the treatment regardless of type of approach, either a two-stage treatment using a Bionator followed by a fixed appliance, or a single-stage treatment using an extraoral appliance combined with a fixed appliance. Correction in a single stage seems to be more efficient, as it requires less time and, therefore, less expensive. A systematic review that evaluated the changes in facial profile after treatment using activators and Bionators found that results are controversial, as the statistically significant data were of questionable clinical significance^40^. 

Studies using CT scans found an increase in airflow in the oropharynx of patients with skeletal Class II malocclusion treated with functional appliances^21^ ^,^ ^23^. These appliances seem to keep the tongue in a more advanced position, which indirectly increases posterior airflow space^21^. However, the radiographs used in both cases described here were two-dimensional, and, therefore, did not show any possible changes in oropharyngeal volume. 

Special circumstances, such as the child’s psychosocial characteristics, risk of accidents and chances of tooth fractures and family preferences, should be taken into account when defining an orthodontic treatment plan.

## CONCLUSION

The treatment of skeletal Class II malocclusion during growth using a Bionator or an extraoral appliance had predictable results, with changes in the facial growth pattern, functional occlusion, long-term stability and satisfactory facial esthetics.
